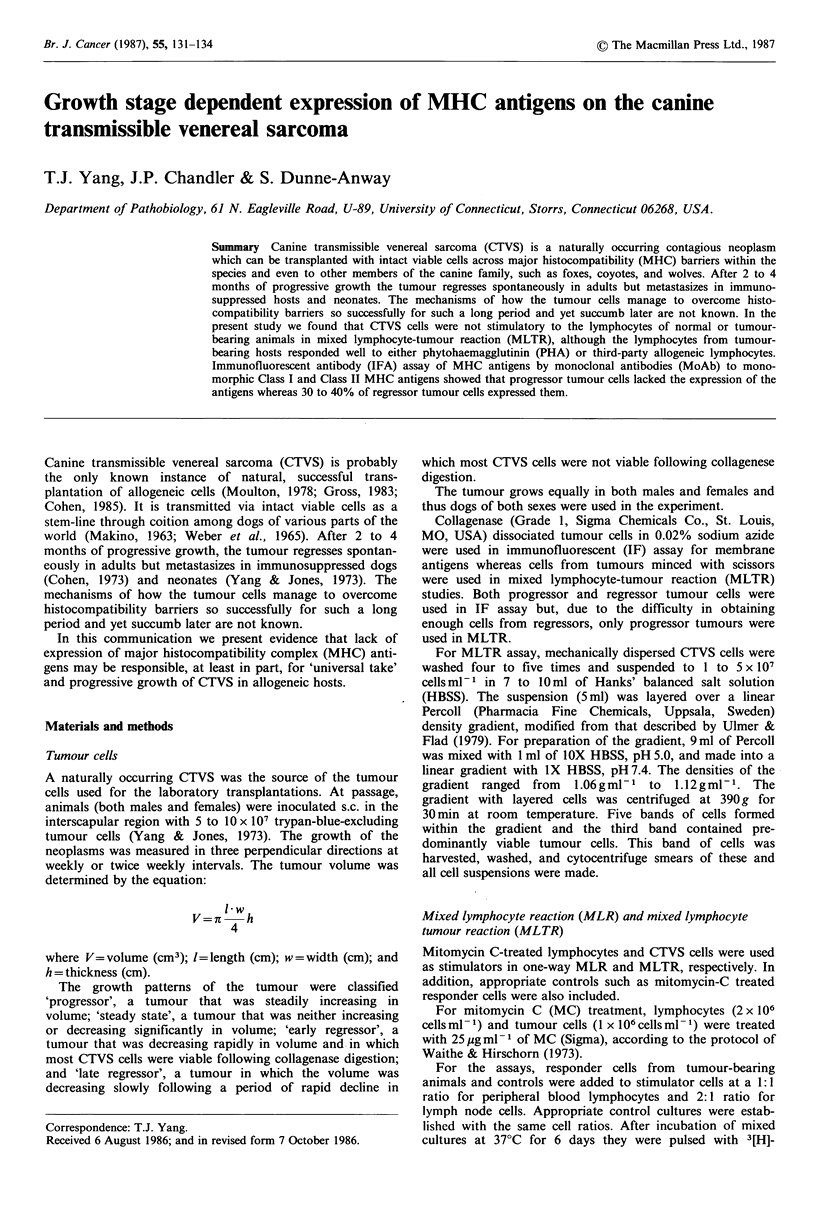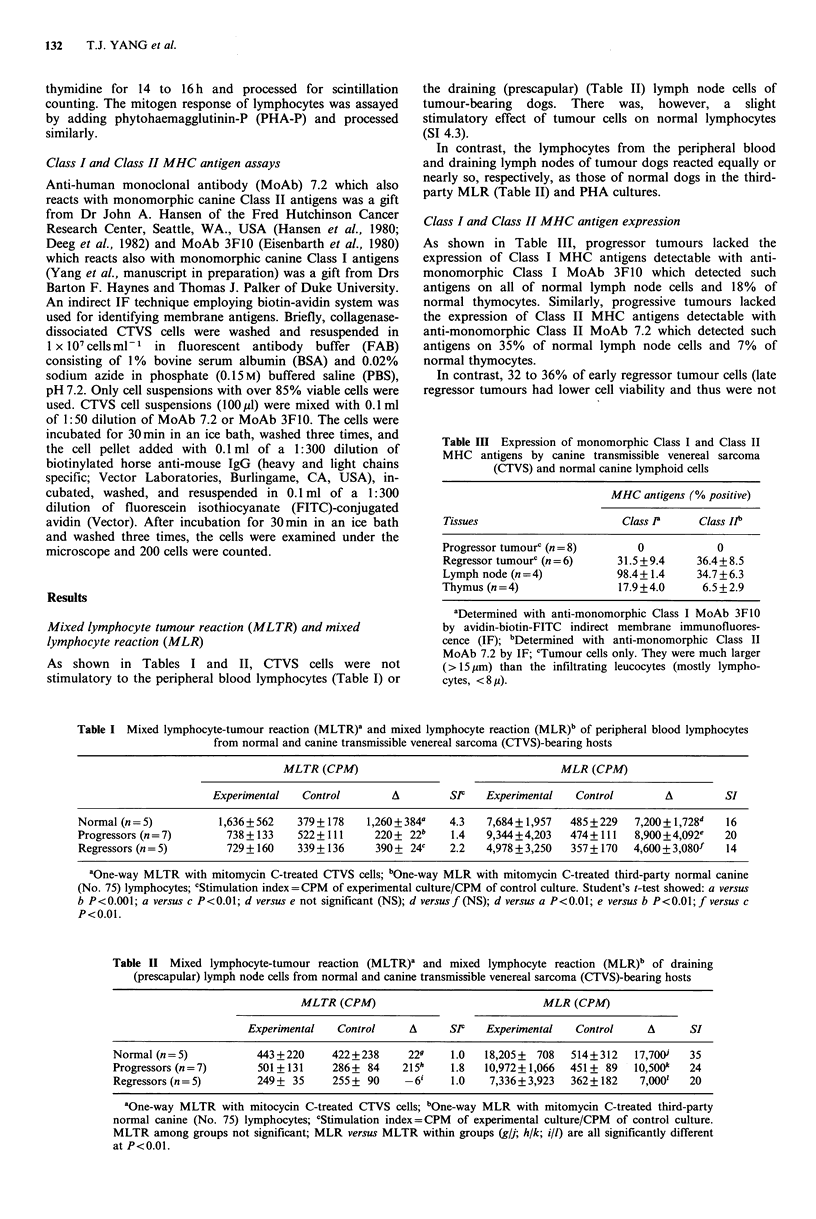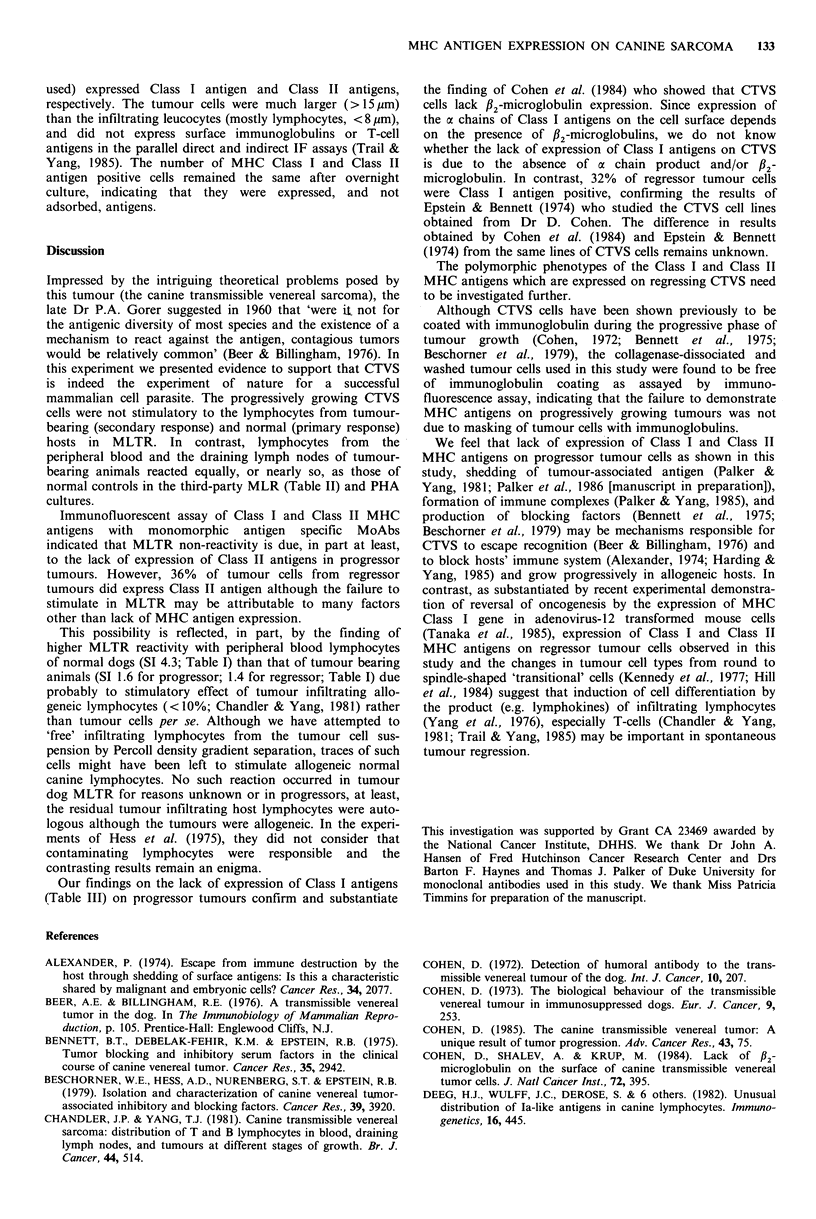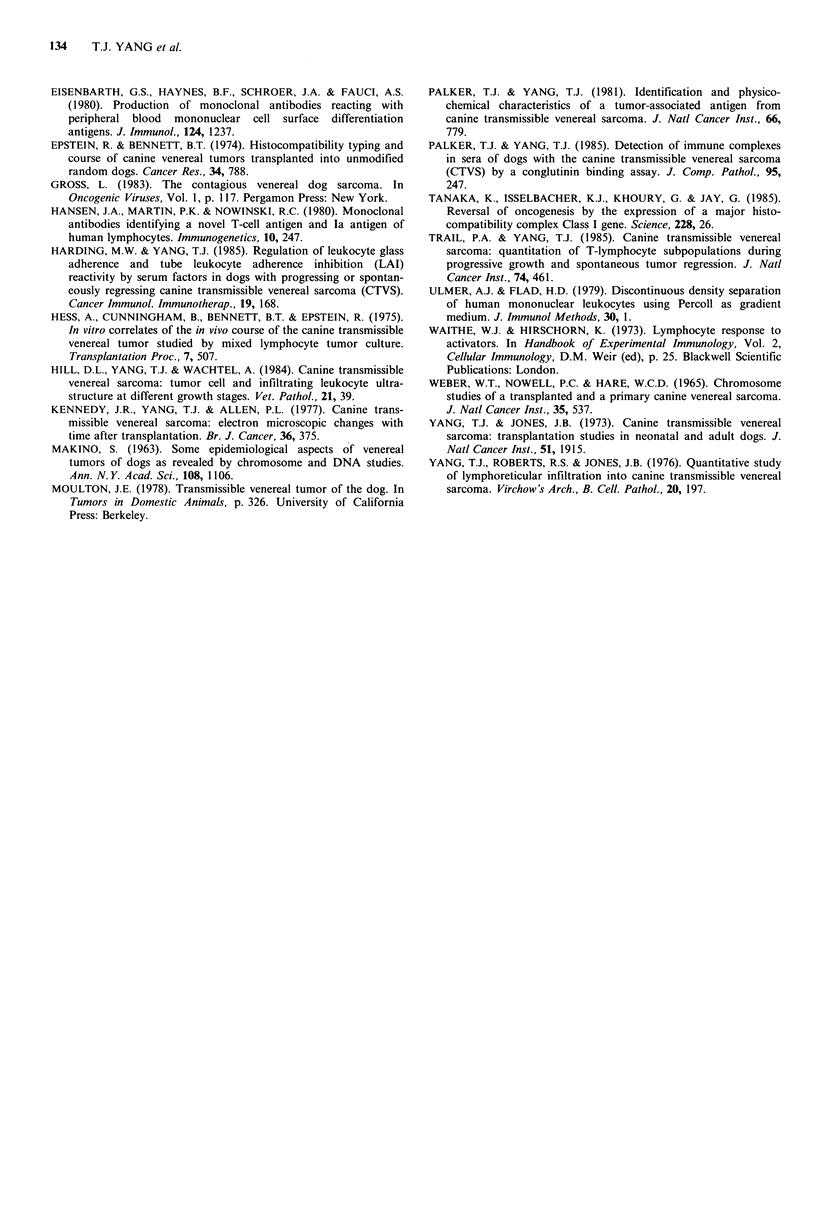# Growth stage dependent expression of MHC antigens on the canine transmissible venereal sarcoma.

**DOI:** 10.1038/bjc.1987.27

**Published:** 1987-02

**Authors:** T. J. Yang, J. P. Chandler, S. Dunne-Anway

## Abstract

Canine transmissible venereal sarcoma (CTVS) is a naturally occurring contagious neoplasm which can be transplanted with intact viable cells across major histocompatibility (MHC) barriers within the species and even to other members of the canine family, such as foxes, coyotes, and wolves. After 2 to 4 months of progressive growth the tumour regresses spontaneously in adults but metastasizes in immunosuppressed hosts and neonates. The mechanisms of how the tumour cells manage to overcome histocompatibility barriers so successfully for such a long period and yet succumb later are not known. In the present study we found that CTVS cells were not stimulatory to the lymphocytes of normal or tumour-bearing animals in mixed lymphocyte-tumour reaction (MLTR), although the lymphocytes from tumour-bearing animals in mixed lymphocyte-tumour reaction (MLTR), although the lymphocytes from tumour-bearing hosts responded well to either phytohaemagglutinin (PHA) or third-party allogeneic lymphocytes. Immunofluorescent antibody (IFA) assay of MHC antigens by monoclonal antibodies (MoAb) to monomorphic Class I and Class II MHC antigens showed that progressor tumour cells lacked the expression of the antigens whereas 30 to 40% of regressor tumour cells expressed them.


*Br. J. Cancer* (1987), **55**, 131–134 © The Macmillan Press Ltd., 1987

### Growth stage dependent expression of MHC antigens on the canine transmissible venereal sarcoma

T.J. Yang, J.P. Chandler & S. Dunne-Anway

*Department of Pathobiology, 61 N. Eagleville Road, U-89, University of Connecticut, Storrs, Connecticut 06268, USA.*

**Summary** Canine transmissible venereal sarcoma (CTVS) is a naturally occurring contagious neoplasm which can be transplanted with intact viable cells across major histocompatibility (MHC) barriers within the species and even to other members of the canine family, such as foxes, coyotes, and wolves. After 2 to 4 months of progressive growth the tumour regresses spontaneously in adults but metastasizes in immunosuppressed hosts and neonates. The mechanisms of how the tumour cells manage to overcome histocompatibility barriers so successfully for such a long period and yet succumb later are not known. In the present study we found that CTVS cells were not stimulatory to the lymphocytes of normal or tumour-bearing animals in mixed lymphocyte-tumour reaction (MLTR), although the lymphocytes from tumour-bearing hosts responded well to either phytohaemagglutinin (PHA) or third-party allogeneic lymphocytes. Immunofluorescent antibody (IFA) assay of MHC antigens by monoclonal antibodies (MoAb) to monomorphic Class I and Class II MHC antigens showed that progressor tumour cells lacked the expression of the antigens whereas 30 to 40% of regressor tumour cells expressed them.

Canine transmissible venereal sarcoma (CTVS) is probably the only known instance of natural, successful transplantation of allogeneic cells (Moulton, 1978; Gross, 1983; Cohen, 1985). It is transmitted via intact viable cells as a stem-line through coition among dogs of various parts of the world (Makino, 1963; Weber *et al.*, 1965). After 2 to 4 months of progressive growth, the tumour regresses spontaneously in adults but metastasizes in immunosuppressed dogs (Cohen, 1973) and neonates (Yang & Jones, 1973). The mechanisms of how the tumour cells manage to overcome histocompatibility barriers so successfully for such a long period and yet succumb later are not known.

In this communication we present evidence that lack of expression of major histocompatibility complex (MHC) antigens may be responsible, at least in part, for 'universal take' and progressive growth of CTVS in allogeneic hosts.

### Materials and methods

### *Tumour cells*

A naturally occurring CTVS was the source of the tumour cells used for the laboratory transplantations. At passage, animals (both males and females) were inoculated s.c. in the interscapular region with 5 to $10 \times 10^7$ trypan-blue-excluding tumour cells (Yang & Jones, 1973). The growth of the neoplasms was measured in three perpendicular directions at weekly or twice weekly intervals. The tumour volume was determined by the equation:

$$V = \pi \frac{l \cdot w}{4} h$$

where $V$ = volume ($cm^3$); $l$ = length (cm); $w$ = width (cm); and $h$ = thickness (cm).

The growth patterns of the tumour were classified 'progressor', a tumour that was steadily increasing in volume; 'steady state', a tumour that was neither increasing or decreasing significantly in volume; 'early regressor', a tumour that was decreasing rapidly in volume and in which most CTVS cells were viable following collagenase digestion; and 'late regressor', a tumour in which the volume was decreasing slowly following a period of rapid decline in which most CTVS cells were not viable following collagenese digestion.

The tumour grows equally in both males and females and thus dogs of both sexes were used in the experiment.

Collagenase (Grade 1, Sigma Chemicals Co., St. Louis, MO, USA) dissociated tumour cells in 0.02% sodium azide were used in immunofluorescent (IF) assay for membrane antigens whereas cells from tumours minced with scissors were used in mixed lymphocyte-tumour reaction (MLTR) studies. Both progressor and regressor tumour cells were used in IF assay but, due to the difficulty in obtaining enough cells from regressors, only progressor tumours were used in MLTR.

For MLTR assay, mechanically dispersed CTVS cells were washed four to five times and suspended to 1 to $5 \times 10^7$ cells $ml^{-1}$ in 7 to 10 ml of Hanks' balanced salt solution (HBSS). The suspension (5 ml) was layered over a linear Percoll (Pharmacia Fine Chemicals, Uppsala, Sweden) density gradient, modified from that described by Ulmer & Flad (1979). For preparation of the gradient, 9 ml of Percoll was mixed with 1 ml of 10X HBSS, pH 5.0, and made into a linear gradient with 1X HBSS, pH 7.4. The densities of the gradient ranged from 1.06 g $ml^{-1}$ to 1.12 g $ml^{-1}$. The gradient with layered cells was centrifuged at 390 *g* for 30 min at room temperature. Five bands of cells formed within the gradient and the third band contained predominantly viable tumour cells. This band of cells was harvested, washed, and cytocentrifuge smears of these and all cell suspensions were made.

### *Mixed lymphocyte reaction (MLR) and mixed lymphocyte tumour reaction (MLTR)*

Mitomycin C-treated lymphocytes and CTVS cells were used as stimulators in one-way MLR and MLTR, respectively. In addition, appropriate controls such as mitomycin-C treated responder cells were also included.

For mitomycin C (MC) treatment, lymphocytes ($2 \times 10^6$ cells $ml^{-1}$) and tumour cells ($1 \times 10^6$ cells $ml^{-1}$) were treated with 25 $\mu g\ ml^{-1}$ of MC (Sigma), according to the protocol of Waithe & Hirschorn (1973).

For the assays, responder cells from tumour-bearing animals and controls were added to stimulator cells at a 1:1 ratio for peripheral blood lymphocytes and 2:1 ratio for lymph node cells. Appropriate control cultures were established with the same cell ratios. After incubation of mixed cultures at 37°C for 6 days they were pulsed with $^3$[H]-

---

Correspondence: T.J. Yang.

Received 6 August 1986; and in revised form 7 October 1986.

thymidine for 14 to 16 h and processed for scintillation counting. The mitogen response of lymphocytes was assayed by adding phytohaemagglutinin-P (PHA-P) and processed similarly.

*Class I and Class II MHC antigen assays*

Anti-human monoclonal antibody (MoAb) 7.2 which also reacts with monomorphic canine Class II antigens was a gift from Dr John A. Hansen of the Fred Hutchinson Cancer Research Center, Seattle, WA., USA (Hansen *et al.*, 1980; Deeg *et al.*, 1982) and MoAb 3F10 (Eisenbarth *et al.*, 1980) which reacts also with monomorphic canine Class I antigens (Yang *et al.*, manuscript in preparation) was a gift from Drs Barton F. Haynes and Thomas J. Palker of Duke University. An indirect IF technique employing biotin-avidin system was used for identifying membrane antigens. Briefly, collagenase-dissociated CTVS cells were washed and resuspended in $1 \times 10^7$ cells $ml^{-1}$ in fluorescent antibody buffer (FAB) consisting of 1% bovine serum albumin (BSA) and 0.02% sodium azide in phosphate (0.15 M) buffered saline (PBS), pH 7.2. Only cell suspensions with over 85% viable cells were used. CTVS cell suspensions (100 $\mu$l) were mixed with 0.1 ml of 1:50 dilution of MoAb 7.2 or MoAb 3F10. The cells were incubated for 30 min in an ice bath, washed three times, and the cell pellet added with 0.1 ml of a 1:300 dilution of biotinylated horse anti-mouse IgG (heavy and light chains specific; Vector Laboratories, Burlingame, CA, USA), incubated, washed, and resuspended in 0.1 ml of a 1:300 dilution of fluorescein isothiocyanate (FITC)-conjugated avidin (Vector). After incubation for 30 min in an ice bath and washed three times, the cells were examined under the microscope and 200 cells were counted.

### Results

*Mixed lymphocyte tumour reaction (MLTR) and mixed lymphocyte reaction (MLR)*

As shown in Tables I and II, CTVS cells were not stimulatory to the peripheral blood lymphocytes (Table I) or the draining (prescapular) (Table II) lymph node cells of tumour-bearing dogs. There was, however, a slight stimulatory effect of tumour cells on normal lymphocytes (SI 4.3).

In contrast, the lymphocytes from the peripheral blood and draining lymph nodes of tumour dogs reacted equally or nearly so, respectively, as those of normal dogs in the third-party MLR (Table II) and PHA cultures.

*Class I and Class II MHC antigen expression*

As shown in Table III, progressor tumours lacked the expression of Class I MHC antigens detectable with anti-monomorphic Class I MoAb 3F10 which detected such antigens on all of normal lymph node cells and 18% of normal thymocytes. Similarly, progressive tumours lacked the expression of Class II MHC antigens detectable with anti-monomorphic Class II MoAb 7.2 which detected such antigens on 35% of normal lymph node cells and 7% of normal thymocytes.

In contrast, 32 to 36% of early regressor tumour cells (late regressor tumours had lower cell viability and thus were not

**Table III** Expression of monomorphic Class I and Class II MHC antigens by canine transmissible venereal sarcoma (CTVS) and normal canine lymphoid cells

| | *MHC antigens (% positive)* | |
|---|---|---|
| *Tissues* | *Class I*[a] | *Class II*[b] |
| Progressor tumour[c] ($n=8$) | 0 | 0 |
| Regressor tumour[c] ($n=6$) | 31.5±9.4 | 36.4±8.5 |
| Lymph node ($n=4$) | 98.4±1.4 | 34.7±6.3 |
| Thymus ($n=4$) | 17.9±4.0 | 6.5±2.9 |

[a]Determined with anti-monomorphic Class I MoAb 3F10 by avidin-biotin-FITC indirect membrane immunofluorescence (IF); [b]Determined with anti-monomorphic Class II MoAb 7.2 by IF; [c]Tumour cells only. They were much larger (>15 $\mu$m) than the infiltrating leucocytes (mostly lymphocytes, <8 $\mu$).

**Table I** Mixed lymphocyte-tumour reaction (MLTR)[a] and mixed lymphocyte reaction (MLR)[b] of peripheral blood lymphocytes from normal and canine transmissible venereal sarcoma (CTVS)-bearing hosts

| | *MLTR (CPM)* | | | | *MLR (CPM)* | | | |
|---|---|---|---|---|---|---|---|---|
| | *Experimental* | *Control* | Δ | *SI*[c] | *Experimental* | *Control* | Δ | *SI* |
| Normal ($n=5$) | 1,636±562 | 379±178 | 1,260±384[a] | 4.3 | 7,684±1,957 | 485±229 | 7,200±1,728[d] | 16 |
| Progressors ($n=7$) | 738±133 | 522±111 | 220± 22[b] | 1.4 | 9,344±4,203 | 474±111 | 8,900±4,092[e] | 20 |
| Regressors ($n=5$) | 729±160 | 339±136 | 390± 24[c] | 2.2 | 4,978±3,250 | 357±170 | 4,600±3,080[f] | 14 |

[a]One-way MLTR with mitomycin C-treated CTVS cells; [b]One-way MLR with mitomycin C-treated third-party normal canine (No. 75) lymphocytes; [c]Stimulation index = CPM of experimental culture/CPM of control culture. Student's $t$-test showed: *a versus b* $P<0.001$; *a versus c* $P<0.01$; *d versus e* not significant (NS); *d versus f* (NS); *d versus a* $P<0.01$; *e versus b* $P<0.01$; *f versus c* $P<0.01$.

**Table II** Mixed lymphocyte-tumour reaction (MLTR)[a] and mixed lymphocyte reaction (MLR)[b] of draining (prescapular) lymph node cells from normal and canine transmissible venereal sarcoma (CTVS)-bearing hosts

| | *MLTR (CPM)* | | | | *MLR (CPM)* | | | |
|---|---|---|---|---|---|---|---|---|
| | *Experimental* | *Control* | Δ | *SI*[c] | *Experimental* | *Control* | Δ | *SI* |
| Normal ($n=5$) | 443±220 | 422±238 | 22[g] | 1.0 | 18,205± 708 | 514±312 | 17,700[j] | 35 |
| Progressors ($n=7$) | 501±131 | 286± 84 | 215[h] | 1.8 | 10,972±1,066 | 451± 89 | 10,500[k] | 24 |
| Regressors ($n=5$) | 249± 35 | 255± 90 | −6[i] | 1.0 | 7,336±3,923 | 362±182 | 7,000[l] | 20 |

[a]One-way MLTR with mitocycin C-treated CTVS cells; [b]One-way MLR with mitomycin C-treated third-party normal canine (No. 75) lymphocytes; [c]Stimulation index = CPM of experimental culture/CPM of control culture. MLTR among groups not significant; MLR *versus* MLTR within groups (*g*/*j*; *h*/*k*; *i*/*l*) are all significantly different at $P<0.01$.

used) expressed Class I antigen and Class II antigens, respectively. The tumour cells were much larger (>15 μm) than the infiltrating leucocytes (mostly lymphocytes, <8 μm), and did not express surface immunoglobulins or T-cell antigens in the parallel direct and indirect IF assays (Trail & Yang, 1985). The number of MHC Class I and Class II antigen positive cells remained the same after overnight culture, indicating that they were expressed, and not adsorbed, antigens.

### Discussion

Impressed by the intriguing theoretical problems posed by this tumour (the canine transmissible venereal sarcoma), the late Dr P.A. Gorer suggested in 1960 that 'were it not for the antigenic diversity of most species and the existence of a mechanism to react against the antigen, contagious tumors would be relatively common' (Beer & Billingham, 1976). In this experiment we presented evidence to support that CTVS is indeed the experiment of nature for a successful mammalian cell parasite. The progressively growing CTVS cells were not stimulatory to the lymphocytes from tumour-bearing (secondary response) and normal (primary response) hosts in MLTR. In contrast, lymphocytes from the peripheral blood and the draining lymph nodes of tumour-bearing animals reacted equally, or nearly so, as those of normal controls in the third-party MLR (Table II) and PHA cultures.

Immunofluorescent assay of Class I and Class II MHC antigens with monomorphic antigen specific MoAbs indicated that MLTR non-reactivity is due, in part at least, to the lack of expression of Class II antigens in progressor tumours. However, 36% of tumour cells from regressor tumours did express Class II antigen although the failure to stimulate in MLTR may be attributable to many factors other than lack of MHC antigen expression.

This possibility is reflected, in part, by the finding of higher MLTR reactivity with peripheral blood lymphocytes of normal dogs (SI 4.3; Table I) than that of tumour bearing animals (SI 1.6 for progressor; 1.4 for regressor; Table I) due probably to stimulatory effect of tumour infiltrating allogeneic lymphocytes (<10%; Chandler & Yang, 1981) rather than tumour cells *per se*. Although we have attempted to 'free' infiltrating lymphocytes from the tumour cell suspension by Percoll density gradient separation, traces of such cells might have been left to stimulate allogeneic normal canine lymphocytes. No such reaction occurred in tumour dog MLTR for reasons unknown or in progressors, at least, the residual tumour infiltrating host lymphocytes were autologous although the tumours were allogeneic. In the experiments of Hess *et al.* (1975), they did not consider that contaminating lymphocytes were responsible and the contrasting results remain an enigma.

Our findings on the lack of expression of Class I antigens (Table III) on progressor tumours confirm and substantiate the finding of Cohen *et al.* (1984) who showed that CTVS cells lack $\beta_2$-microglobulin expression. Since expression of the $\alpha$ chains of Class I antigens on the cell surface depends on the presence of $\beta_2$-microglobulins, we do not know whether the lack of expression of Class I antigens on CTVS is due to the absence of $\alpha$ chain product and/or $\beta_2$-microglobulin. In contrast, 32% of regressor tumour cells were Class I antigen positive, confirming the results of Epstein & Bennett (1974) who studied the CTVS cell lines obtained from Dr D. Cohen. The difference in results obtained by Cohen *et al.* (1984) and Epstein & Bennett (1974) from the same lines of CTVS cells remains unknown.

The polymorphic phenotypes of the Class I and Class II MHC antigens which are expressed on regressing CTVS need to be investigated further.

Although CTVS cells have been shown previously to be coated with immunoglobulin during the progressive phase of tumour growth (Cohen, 1972; Bennett *et al.*, 1975; Beschorner *et al.*, 1979), the collagenase-dissociated and washed tumour cells used in this study were found to be free of immunoglobulin coating as assayed by immunofluorescence assay, indicating that the failure to demonstrate MHC antigens on progressively growing tumours was not due to masking of tumour cells with immunoglobulins.

We feel that lack of expression of Class I and Class II MHC antigens on progressor tumour cells as shown in this study, shedding of tumour-associated antigen (Palker & Yang, 1981; Palker *et al.*, 1986 [manuscript in preparation]), formation of immune complexes (Palker & Yang, 1985), and production of blocking factors (Bennett *et al.*, 1975; Beschorner *et al.*, 1979) may be mechanisms responsible for CTVS to escape recognition (Beer & Billingham, 1976) and to block hosts' immune system (Alexander, 1974; Harding & Yang, 1985) and grow progressively in allogeneic hosts. In contrast, as substantiated by recent experimental demonstration of reversal of oncogenesis by the expression of MHC Class I gene in adenovirus-12 transformed mouse cells (Tanaka *et al.*, 1985), expression of Class I and Class II MHC antigens on regressor tumour cells observed in this study and the changes in tumour cell types from round to spindle-shaped 'transitional' cells (Kennedy *et al.*, 1977; Hill *et al.*, 1984) suggest that induction of cell differentiation by the product (e.g. lymphokines) of infiltrating lymphocytes (Yang *et al.*, 1976), especially T-cells (Chandler & Yang, 1981; Trail & Yang, 1985) may be important in spontaneous tumour regression.

This investigation was supported by Grant CA 23469 awarded by the National Cancer Institute, DHHS. We thank Dr John A. Hansen of Fred Hutchinson Cancer Research Center and Drs Barton F. Haynes and Thomas J. Palker of Duke University for monoclonal antibodies used in this study. We thank Miss Patricia Timmins for preparation of the manuscript.

### References

ALEXANDER, P. (1974). Escape from immune destruction by the host through shedding of surface antigens: Is this a characteristic shared by malignant and embryonic cells? *Cancer Res.*, **34**, 2077.

BEER, A.E. & BILLINGHAM, R.E. (1976). A transmissible venereal tumor in the dog. In *The Immunobiology of Mammalian Reproduction*, p. 105. Prentice-Hall: Englewood Cliffs, N.J.

BENNETT, B.T., DEBELAK-FEHIR, K.M. & EPSTEIN, R.B. (1975). Tumor blocking and inhibitory serum factors in the clinical course of canine venereal tumor. *Cancer Res.*, **35**, 2942.

BESCHORNER, W.E., HESS, A.D., NURENBERG, S.T. & EPSTEIN, R.B. (1979). Isolation and characterization of canine venereal tumor-associated inhibitory and blocking factors. *Cancer Res.*, **39**, 3920.

CHANDLER, J.P. & YANG, T.J. (1981). Canine transmissible venereal sarcoma: distribution of T and B lymphocytes in blood, draining lymph nodes, and tumours at different stages of growth. *Br. J. Cancer*, **44**, 514.

COHEN, D. (1972). Detection of humoral antibody to the transmissible venereal tumour of the dog. *Int. J. Cancer*, **10**, 207.

COHEN, D. (1973). The biological behaviour of the transmissible venereal tumour in immunosuppressed dogs. *Eur. J. Cancer*, **9**, 253.

COHEN, D. (1985). The canine transmissible venereal tumor: A unique result of tumor progression. *Adv. Cancer Res.*, **43**, 75.

COHEN, D., SHALEV, A. & KRUP, M. (1984). Lack of $\beta_2$-microglobulin on the surface of canine transmissible venereal tumor cells. *J. Natl Cancer Inst.*, **72**, 395.

DEEG, H.J., WULFF, J.C., DEROSE, S. & 6 others. (1982). Unusual distribution of Ia-like antigens in canine lymphocytes. *Immunogenetics*, **16**, 445.

EISENBARTH, G.S., HAYNES, B.F., SCHROER, J.A. & FAUCI, A.S. (1980). Production of monoclonal antibodies reacting with peripheral blood mononuclear cell surface differentiation antigens. *J. Immunol.*, **124**, 1237.

EPSTEIN, R. & BENNETT, B.T. (1974). Histocompatibility typing and course of canine venereal tumors transplanted into unmodified random dogs. *Cancer Res.*, **34**, 788.

GROSS, L. (1983). The contagious venereal dog sarcoma. In *Oncogenic Viruses*, Vol. 1, p. 117. Pergamon Press: New York.

HANSEN, J.A., MARTIN, P.K. & NOWINSKI, R.C. (1980). Monoclonal antibodies identifying a novel T-cell antigen and Ia antigen of human lymphocytes. *Immunogenetics*, **10**, 247.

HARDING, M.W. & YANG, T.J. (1985). Regulation of leukocyte glass adherence and tube leukocyte adherence inhibition (LAI) reactivity by serum factors in dogs with progressing or spontaneously regressing canine transmissible venereal sarcoma (CTVS). *Cancer Immunol. Immunotherap.*, **19**, 168.

HESS, A., CUNNINGHAM, B., BENNETT, B.T. & EPSTEIN, R. (1975). *In vitro* correlates of the *in vivo* course of the canine transmissible venereal tumor studied by mixed lymphocyte tumor culture. *Transplantation Proc.*, **7**, 507.

HILL, D.L., YANG, T.J. & WACHTEL, A. (1984). Canine transmissible venereal sarcoma: tumor cell and infiltrating leukocyte ultrastructure at different growth stages. *Vet. Pathol.*, **21**, 39.

KENNEDY, J.R., YANG, T.J. & ALLEN, P.L. (1977). Canine transmissible venereal sarcoma: electron microscopic changes with time after transplantation. *Br. J. Cancer*, **36**, 375.

MAKINO, S. (1963). Some epidemiological aspects of venereal tumors of dogs as revealed by chromosome and DNA studies. *Ann. N.Y. Acad. Sci.*, **108**, 1106.

MOULTON, J.E. (1978). Transmissible venereal tumor of the dog. In *Tumors in Domestic Animals*, p. 326. University of California Press: Berkeley.

PALKER, T.J. & YANG, T.J. (1981). Identification and physicochemical characteristics of a tumor-associated antigen from canine transmissible venereal sarcoma. *J. Natl Cancer Inst.*, **66**, 779.

PALKER, T.J. & YANG, T.J. (1985). Detection of immune complexes in sera of dogs with the canine transmissible venereal sarcoma (CTVS) by a conglutinin binding assay. *J. Comp. Pathol.*, **95**, 247.

TANAKA, K., ISSELBACHER, K.J., KHOURY, G. & JAY, G. (1985). Reversal of oncogenesis by the expression of a major histocompatibility complex Class I gene. *Science*, **228**, 26.

TRAIL, P.A. & YANG, T.J. (1985). Canine transmissible venereal sarcoma: quantitation of T-lymphocyte subpopulations during progressive growth and spontaneous tumor regression. *J. Natl Cancer Inst.*, **74**, 461.

ULMER, A.J. & FLAD, H.D. (1979). Discontinuous density separation of human mononuclear leukocytes using Percoll as gradient medium. *J. Immunol Methods*, **30**, 1.

WAITHE, W.J. & HIRSCHORN, K. (1973). Lymphocyte response to activators. In *Handbook of Experimental Immunology*, Vol. 2, *Cellular Immunology*, D.M. Weir (ed), p. 25. Blackwell Scientific Publications: London.

WEBER, W.T., NOWELL, P.C. & HARE, W.C.D. (1965). Chromosome studies of a transplanted and a primary canine venereal sarcoma. *J. Natl Cancer Inst.*, **35**, 537.

YANG, T.J. & JONES, J.B. (1973). Canine transmissible venereal sarcoma: transplantation studies in neonatal and adult dogs. *J. Natl Cancer Inst.*, **51**, 1915.

YANG, T.J., ROBERTS, R.S. & JONES, J.B. (1976). Quantitative study of lymphoreticular infiltration into canine transmissible venereal sarcoma. *Virchow's Arch., B. Cell. Pathol.*, **20**, 197.